# Valedictory Address on Behalf of the Faculty to the Graduating Class in Atlanta Medical College, at the Annual Commencement, on Friday, August 30, 1867

**Published:** 1867-09

**Authors:** J. M. Johnson


ATLANTA

Medical and Surgical Journal.

NEW SERIES.

Vol. VIII. SEPTEMBER, 1867. No. 7.

### ORIGINAL COMMUNICATIONS.

### ARTICLE I.

*Valedictory Address on behalf of the Faculty to the Graduating Class in Atlanta Medical College, at the Annual Commencement, on Friday, August* 30, 1867. By J. M. Johnson, M. D.

Gentlemen of the Graduating Class.

I have been honored with an invitation from your distinguished teachers, to address you on this interesting occasion. It is the most important one of your lives, because you go forth under the sanction of the laws of Georgia, as Doctors of Medicine, prepared to commence the practice of the Healing Art; and for this purpose, and to this extent, you are recommended to the people, and to the brotherhood of medicine wherever the liberal sciences are taught. You go forth, therefore, from your Alma Mater, crowned with the best wishes and highest hopes of your teachers, the board of trustees, and the citizens of Atlanta. And in the name of each and every one, I tender you an earnest God-speed in the battle of life you are about to begin.

The dream of age, is, when I was a boy. The dream of youth, when shall I be a man. Many, in the sear and yellow leaf, looking back to the period of life from which you start to-day, would be but too happy, were it possible to begin life anew, so that profiting by their failures, and perhaps their errors and mistakes, they might add to their own happiness and that of others. But it is a well received maxim, that we are wise too late. The thoughtlessness of youth prompts the belief, that in attaining to manhood, the *ultima thule* of desire with the young, perennial flowers will cover his pathway, and new beauties and new charms regale him at every step. This delusion is dispelled by the stern events that meet him everywhere along the path of manhood. From to-day you begin to drink the bitter waters of experience in the capacity of practitioners of medicine. Hard and laborious as have been your struggles to meet the responsibilities of this hour, it will require harder and more laborious efforts still to fill, with credit to yourselves and benefit to your patrons, the new role in which your love of science calls you to act. Every friend of science must expect to be laughed at and even aspersed by the foolish, vain-glorious, and jealous. Such is the history of the past: A Carthagenian boy, at the age of sixteen years, laid his hand upon his country's altar and swore eternal enmity to Rome. He marched his army to her walls, and held her country for seventeen years. His sublime resolve placed him above the adulation of friends and the taunts of enemies.

If you will enter upon your career with the sublime inspiration that earnestness of purpose always carries with it, you will be an honor to your distinguished faculty, to your country, and to mankind. Under this pledge, which the acceptance of your diplomas leads me to infer, permit me to offer you a few thoughts on

LIFE—ITS OBJECT.

So vast are the ramifications of my subject, that I approach it with unaffected diffidence; nor can I hope to rise to the "height of the great argument,"

Man is so variously and so wonderfully endowed, approximating angels in virtue, and demons in vice and crime, that but for ethical demonstration to the contrary, it would appear that fate had irrevocably fixed his destiny. But our system of education, imperfect as it is, and our holy religion, mingled, as is too often the case, with hypocricy and infidelity; our pilosophy tortured for convenience by prurient theorists from its rational teachings,—all demonstrate the perfectibility of human nature to the point where infinite love imputes the righteousness of the vicarious atonement, and transforms it from corruption to incorruption. We, therefore, assume that life has been given for an object, and that object, continual progression.

Life is made up of functions, which, in this connection, we apply to the body; of faculties, which we apply to the mind; and powers, which we apply to the soul. The body is a mass of matter, and without these co-existing principles, would fall into immediate decay; but with these manifestations, it is a grand whole, and capable of indefinite progression.

The body, directed by the mind, looks to all the contingencies that may surround it. It provides for day and night, summer and winter, sickness and health; seeks refreshment in food and drink, and restoration from toil and anxiety, in sleep; provides for its safety in the face of danger, cultivates an agreeable relation with every thing around it; moves in the graceful dance; from the little colony, it peoples a continent, fells the sturdy oak, plants the vine and orchard, subdues the wilderness, ploughs the ocean, scales mountains, annihilates distance, carries burdens, builds cities, organizes governments, erects monuments to perpetuate its glory,—and dies. This is the influence of mind over matters. Thus are its faculties excited, to exalt and adorn the rude abode assigned it by the great Master, and over which it presides, and which it controls. But I hear the inquiry, does mind also prompt to evil deeds, and deform and debase the picture you have drawn by the blackest crimes? I answer, unhesitatingly, yes. I have the belief, and I state it fearlessly, but with all due reverence, that the soul or spirit comes from God, and takes up its abode with every young life, and by its contact, makes the intellect or mind immortal, and becomes the imbodiment of the virtues of the good man, and finally his entity. But to him that fails to obey the great law of progress, or who falls by the way, it stamps the mind with immortality, and gives place to the fiend of darkness, which becomes his person and entity. The rejected soul, or spirit, thenceforth becomes an accuser and witness, and no traverse can impair his testimony; for the "blood of the covenant has been accounted an unclean thing."

Hence, the varied phases of human character, condition, and crime. Here it weeps with misfortune; there it strikes the assassin's blow: in one instance it exalts to heaven—in another it drags down to hell; brother lifts his hand against brother, father against son; kindness and cruelty, blessing and cursing. These are the scenes of every day life. But no matter what the body may do, it has been impelled by that faculty we call the mind, to the execution of it.

But the mind is also a pupil, and is under the direct guardianship of the soul or spirit. As soon as the period of adolescence dawns, the soul prompts the mind to inquire, Whence am I? What are the relations I bear to every thing around me? Who is God, and where is his habitation? Metaphysics, the starting point of knowledge, is the first study of the infant mind. Alas, that it should be so soon abandoned! and the little philosopher turned into the street to become a hardened adept in petty crimes, and mature for the pillory or prison.

These inquiries are the promptings of the immortal spirit; and this the foundation from which man must begin to ascend in the scale of morality and virtue. Passion and appetite, falsehood and deception, are the germs of moral disease that must be checked before it mars and deforms and blights the immortal gem. The remedy for all this is found in Metaphysical Science. So simple and easy are its primitive lessons, that even the infant mind can grasp them; and yet so grand in its final results, that the great Newton exclaimed, that after all he had achieved in philosophy, he was but gathering pebbles on the shore of the great ocean of truth.

A system of perceptive education, with physics as a foundation, and taught orally, as the young mind could comprehend it, would direct it into the beautiful regions of philosophy, where truth flows in from perennial streams, and fills each blank in the young mind with lively images; typical of the great future to be opened in the different phazes of natural and spiritual life through which he must assuredly pass. The neglect of a system of didactics, of which the above is not even an outline, you will learn in the progress of your experience, is the great crime of society and of States.

I am no casuist; nor do I propose to meddle with other peoples' consciences. But the glory of the primitive ages, as seen in the light of history, was their love of literature, of science, and the arts. Four hundred and fifty years before Christ, Philosophy, Poetry, Medicine, Architecture, Painting, and statuary were taught at Athens, Alexandria, and Rome. More than twenty-three centuries have passed, and yet Socrates and Plato, Hippocrates and Aristotle, masters then, are masters now. Thirty centuries ago, Homer sung of the siege and fall of Troy, in immortal verse. The genius of poetry inspired by Marathon, Lodi, and Waterloo; by Cæsar, Washington, and Napoleon; by the rise and fall of empires, peoples, and civilizations,—has scarcely brought forth a rival in Epic Verse. The text-book of Milton, Dryden, and Pope, confounding reviewers and critics, will hold the place for ages to come; it has held for centuries past. Painting is traced into the mythical ages, until inquiry is lost in the ruins of forgotten systems and peoples, and yet the great Angelo called them masters.

All these things live in our museums and libraries, whilst the mind universal is seduced from this grand center to the perephery, in the pursuit of materialistic objects, until one only purpose survives, to which all others are ends. The literature of our day is a whited sepulcher. In this department of human progress, genius, with its vastly increased facilities, has achieved but little. The art of printing has multiplied books; but in literature, with some exceptions, the development of great ideas has not been in proportion to materialistic inventions, to which object the genius of ages has been stimulated, and which has taken the lead in every department of life and business. As a consequence, infidelity fills your pulpits, congregations, and thoroughfares; mammon, and not humility, constitutes the sinews of the church, as it does the beginning and ending of all other pursuits. Rail roads, steam ships, and telegraphs, have opened wide the avenues of commerce, stimulated speculation and traffic, until the world is little less than a theater for trespassers, adventurers, and hucksters. In our own country, the virtues developed by our institutions, it would seem, had perished with the great men who originated them. And we have bartered virtue for ambition, principle for expediency, liberty for despotism.

It has been beautifully said, "gold frits to dust, time rots the diamond"—even the worlds that "darkle" in the trackless void must perish. But the soul, sublime in its aspirations to behold the primeval glories of the burning throne, or in its capacity to endure the depths of black despair, must cleave eternity forever, where is kept no note of time. The great purpose of life is to escape the one and reach the other. And as the body is dependent upon the mind for every excellence that appertains to it; and as the mind is dependent upon the soul, or spirit, for the conception of its duties here and its destiny hereafter; so, in like manner, the soul, or spirit, looks up to the source of all true knowledge and perfection, and brings to him that hungers and thirsts, food such as the angels eat. Mental and moral philosophy, or those branches of philosophy known as metaphysics, and which comprehend the science of the principles of all things existing—including, also, all the phenomena of mind and intelligence—as we have said before, is the alpha and omega of true mortal existence. Aided by the golden pages of revealed wisdom and love to guide your feet in the path of humility and duty, the object of life will be plain. But I warn you against perversions of both. Ingenious sophists abound every where, who, taking reason as their guide, deny every thing that reason cannot comprehend and explain. I need not warn you against this monstrous assumption. Reason cannot guide you in the things that relate to God, if you travel without the pale of revelation. Reason must be limited by facts—all beyond is speculation. The Bible has passed through centuries of criticism: it has been assailed by philosophers, historians, and poets; learning has wasted its treasures to confute it; wits and acrobats have ridiculed it; over-zealous, as well as unfaithful friends have brought reproach upon it,—and yet it lives, and it will continue to grow brighter until the perfect day; for it is the foundation of all government and all law—humane and divine. It has taken away the prerogative of Kings, and established the bill of rights, which even despots cannot assail with impunity. The beggar in his rags, or Dives in his purple, are equals before its sublime precepts. Whereever its empire has been established, altars and gods have fallen before it—and man walks forth redeemed from idolatry into the pure light of faith and love.

But, to conclude this branch of my subject, the great object of life is to be good, and wise, and useful. Ignorance is a great moral crime. The man who is wilfully ignorant, is the greatest of offenders: he offends against himself, society, and his God. Mind has been given him, that he may cultivate and adorn it. In it is gold, and silver, and diamonds; in it is honor and power; in it is present and future happiness. Then lag not behind—let there be no blanks in the noble tablet; but write immortality on every page.

The occasion that has brought me before you to-day, will probably be forgotten by most of this audience in a short time. Such pageants have their importance, nevertheless; and if only our mind is impressed, and one truth fastened, it may prove a telling result. Nothing truly noble can be accomplished without earnestness in thought, in action, and in will. Such men leave their impress upon the world; and it is to this that we are indebted for the progress that has been wrought out, beginning with the Genesis of creation and coming down to our day. Chance has its uses and results, good and bad, but it is blind; and he that depends upon luck for fortune or fame, is blind also. If attained in this way, it is generally a jewel in the wrong place.

All avocations, to be successful, must be directed by courage, earnestness, and a degree, at least, of practical wisdom. But to be a physician, in the true sense of the word, requires the very perfection of these qualities. I have seen farmers who planted imperfectly, and who scarcely cultivated at all, through the fertility of the soil and genial seasons, gather a plentiful harvest. I have seen the same kind of cultivation bring starvation to the door, under less favorable seasons; while the more industrious husbandman, under like circumstances, gleaned plentifully, because he sowed and cultivated with care. The physician who imitates the former, may not go without patronage; but he will never be the man upon whom a whole community will rely, or be willing to trust in the trying emergency of life or death. Such a man should abandon his profession, and engage in any thing else that will call forth all his powers. But what ought to be said of him to whom capacity has been given; whose mind has been cultivated and enlightened by education; who has drank to fruition of the pure waters of science; who has been vouched for by his teachers and professors, and recommended by them for the honors of graduation,—and who, on receiving his diploma, pledges himself to honor it, and thereby do honor to the authority by which it is conferred—and, in the face of all this, falsifies his vows, grows weary of study, tires with the wholesome restraints imposed by conscience and duty, and seeing in his profession only the means for the accomplishment of ends, turns his back upon those who have so earnestly sought his elevation, prostitutes his high calling to the base purpose of quackery, deception and crime! Such a man, we say, unfit to be trusted or countenanced, deserves to have the mark of Cain upon his forehead.

In the prosecution of the future of your professional lives, think not of inglorious ease. The enemies you are called to combat, are neither weak nor few; and they make their assaults at all hours, and in all weather. You may be a preferred guest at the feast; but if the enemy invades your dominion, you must forego the joys of re-union, to meet and despoil him of his conquest. Weary and troubled, you may have thrown your head on the pillow to find in sleep the restoration you so much need, when again you are confronted by a distressed husband or father, who comes to you as his only earthly hope, and pleads not in vain, I trust, the timely interposition of your skill in saving, under Providence, the lives of those dear to him. But other thorns than those resulting from abnegation and fatigue may lacerate your feelings and mortify your honest pride. There is an old vulgarism which, alas, you will find but too true: "no man can please everybody." When you have done all you can, and the best you can, and perhaps the very best that human agency could achieve, the viper will hiss at your feet, and the raven croak from the house top. Jealousy, envy, and malice, will startle you with its tongue of poison. It may even unsettle the confidence of your friends, and fill them, also, with distrust.

There is but one safeguard against this, and that is found in constant vigilence—a vigilence that never tires. You must be not only what your diploma imports, a medical philosopher, but you must be thoroughly acquainted with the opinions of the fathers of the profession, and of your cotemporaries also. In the battle of life, which from to-day you begin, you are sure to fall bleeding and helpless, if you undervalue the ability or shrewdness of your adversary. The universality of the literature of medicine, its accepted truths, founded on the experience of wise men, and faithfully preserved to our own time, will be to you a pillar of cloud by day and of fire in the dark hours of calumny or neglect. To this earnest study, if you add purity of character and a sacred regard for truth, the world will appreciate and honor you. But if addicted to the folly of boasting and exageration, intemperance, gaming, or neglect of business, your enemies will strike through all these, and even your best friends will not be able to defend you.

After all, you must look within your own hearts and to your own consciences for your reward. You will surely never grow rich by the practice of your profession. I need not attempt to prove this,—it is patent to every body. For this, the world will give you no credit, but make it a part of your condemnation, that you have charged high, wasted your money, and died poor, because you have not flaunted your charities, as some do, in the faces of all men. You must be a perpetual tax-payer and contributor, to the very public that thus asperse you. You will help to build up churches and schools, take a leading part in all public charities, and in addition, give full one half of all you make, in time, services, and money, to the poor; and after thus contributing, find yourselves taxed by Corporations and States, as though your profession was a trade. You will be exposed to contagion and pestilence. Public opinion will brand you with desertion and cowardice, if you flee the post of danger. Therefore, you must face the enemies of health and life, no matter under what guise they come; and if you fall a victim, content yourself with being forgotten, except by those who love you, before the first decade has passed.

In entering upon the duties, now newly opening before you, bear in mind what I am about to say, and carry with you throughout your lives these words of solemn truth. Labor, privation, poverty, martyrdom ; some one of these hard conditions, and perhaps all of them, is sure to be your lot, as they have been the lot of your predecessors for more than two thousand years. They founded an art that has done more to bless mankind than all the discoveries of human genius beside, and found their reward in the good they have achieved for suffering humanity. Like them, you must bide your time in this world, and look for your reward where alone duty performed can be properly, and will be surely, rewarded. With all the lights before me, it is difficult to say what are the motives that impell men to the study and practice of medicine, other than the most exalted known to man. Money-making is the passion of man, with rare exceptions. For this they dare everything. Even family, friendship and reputation are cast aside by the votaries of the strong box; and every man's importance is measured by his purse. The still small voice that appeals from the inner temple, warning us of the danger of riches, stifled at first, is finally put to flight, and with its going, the last touch of sympathy is dried up, and the last noble emotion of the soul perishes forever. I need not warn you to beware of riches; for there is no margin in the profession you have chosen, to nurse and cultivate a love of them. You are actuated, I trust, by higher motives. The kindlier and holier feelings of the human heart must be kept constantly in the ascendency, if you would hand your names down to posterity, enrolled high on the list of benefactors of mankind. Strive, therefore, to deserve a just fame, won in the cause of humanity; and at the close of your lives you can say, with the inspired man, "I have fought a good fight."

One word to my fair friends before I close. It is no idle eulogy to say of woman, that she is "Heaven's best gift to man." The first to sin, she has been the first to atone; and her atonement has won for her the title of "Mother of the Son of God." In Eden she was covered with shame. As the mother of nations, she has clothed her children with honor; and in her exaltation, she will appear in heaven, clothed with the sun. No abasement so deep, no atonement so complete, and no exaltation so glorious. Behold the pageant before you to-day—this is your achievement; look at the world's civilization—this you have won from barbarism; man, the slayer of his brother, now meekly kneels with you at the altar of prayer; led by your suffering, gentleness, and virtue, he casts himself at your feet, exalts you from the condition of a slave to a help-mate at his side: and immortality is the prize for both. Go on in your career; pause not before obstacles; your work will not be completed, until poverty and crime, hunger and nakedness, sin and death, are banished from the world: then will your "conquests be borne through the emerald gates of the new Jerusalem, and laid at the feet of the immortal King."

